# Estimating the heritability of psychological measures in the Human Connectome Project dataset

**DOI:** 10.1371/journal.pone.0235860

**Published:** 2020-07-09

**Authors:** Yanting Han, Ralph Adolphs

**Affiliations:** 1 Division of Biology and Biological Engineering, California Institute of Technology, Pasadena, CA, United States of America; 2 Division of the Humanities and Social Sciences, California Institute of Technology, Pasadena, CA, United States of America; 3 Chen Neuroscience Institute, California Institute of Technology, Pasadena, CA, United States of America; University of North Carolina at Chapel Hill, UNITED STATES

## Abstract

The Human Connectome Project (HCP) is a large structural and functional MRI dataset with a rich array of behavioral and genotypic measures, as well as a biologically verified family structure. This makes it a valuable resource for investigating questions about individual differences, including questions about heritability. While its MRI data have been analyzed extensively in this regard, to our knowledge a comprehensive estimation of the heritability of the behavioral dataset has never been conducted. Using a set of behavioral measures of personality, emotion and cognition, we show that it is possible to re-identify the same individual across two testing times (fingerprinting), and to identify identical twins significantly above chance. Standard heritability estimates of 37 behavioral measures were derived from twin correlations, and machine-learning models (univariate linear model, Ridge classifier and Random Forest model) were trained to classify monozygotic twins and dizygotic twins. Correlations between the standard heritability metric and each set of model weights ranged from 0.36 to 0.7, and questionnaire-based and task-based measures did not differ significantly in their heritability. We further explored the heritability of a smaller number of latent factors extracted from the 37 measures and repeated the heritability estimation; in this case, the correlations between the standard heritability and each set of model weights were lower, ranging from 0.05 to 0.43. One specific discrepancy arose for the general intelligence factor, which all models assigned high importance, but the standard heritability calculation did not. We present a thorough investigation of the heritabilities of the behavioral measures in the HCP as a resource for other investigators, and illustrate the utility of machine-learning methods for qualitative characterization of the differential heritability across diverse measures.

## Introduction

With the recent availability of cost-efficient methods for obtaining genomic and neuroimaging data, a number of influential projects have generated large-sample databases that combine multiple types of data across the same set of individuals. For instance, the Nathan Kline Institute database provides physiological, psychological, genetic and neuroimaging data, the Cambridge Center for Aging Neuroscience database provides physiological, cognitive and imaging data, and the UK Biobank provides genotyping, imaging and questionnaire data. Perhaps most widely studied to date is the Human Connectome Project (HCP), an NIH-funded database that offers a uniquely rich sample of measures across the same 1200 subjects: structural, diffusion, and functional MRI, together with questionnaire- and task-based measures that assess many different psychological domains as well as genomic information [1]. This database has been analyzed in hundreds of publications, and is being expanded to include younger age ranges. The HCP dataset has proven to be a valuable resource for investigating individual differences. A number of recent studies have utilized the HCP dataset to predict personal identity, gender, fluid intelligence, personality, and executive function from brain connectivity [2–5]. Another valuable aspect of the HCP is that it has a biologically verified family structure, including 149 genetically confirmed monozygotic twin pairs and 94 genetically confirmed dizygotic twin pairs. Given all the other rich measures in the HCP, this family structure provides an important opportunity for investigating heritability across behavior, brain structure, and brain function. Yet to date no study has characterized the heritability of the diverse behavioral measures available in the HCP’s 1200 subjects, limiting this line of investigation. Our goal in the present study was to provide such a characterization.

Decades of research have accumulated abundant knowledge on the heritability of various human traits. A recent meta-analysis studied 28 functional domains and found the largest heritability estimates for several physical trait domains (such as the ophthalmologic and skeletal domains) but the lowest heritability for some psychological domains (such as the social values domain; [6]). This domain-wise characterization was largely consistent with reported values from studies that focused on individual traits. For example, height is one of the most studied traits in the physical domain. An earlier study involving twins from eight countries estimated the heritability of height to be 0.87–0.93 for males and 0.68–0.84 for females [7], although a more recent study of larger samples produced estimates up to 0.83 in boys and 0.76 in girls [8], comparable to the reported meta heritability of 0.73 [6]. By contrast, the heritability of psychological traits is generally estimated to be lower: episodic memory has a heritability around 0.3–0.6 [9] (with meta heritability around 0.6), and personality has a heritability around 0.4 [10] (with meta heritability around 0.48). These traits have typically been studied in isolation in previous studies. Here we took advantage of the comprehensive set of measures available in the Human Connectome Project (HCP) dataset (including both self-report questionnaires and behavioral tasks), which allowed us to describe an individual’s psychological profile and similarity to others. Our goal was to provide comprehensive estimation of the heritabilities of behavioral measures in this dataset as a resource that could be used for studies of heritability in the neuroimaging data component.

Several studies have used MRI data in the HCP to investigate the heritability of brain structures and connectivity patterns, many aspects of which are heritable [11]. For instance, surface area and cortical thickness [12], the depth of Sulcal Pits [13], subcortical shape [14], hippocampal subfield volumes [15] and cortical myelination [16] are all heritable structural features. Similarly, connectivity patterns, especially resting-state fMRI, have been shown to be heritable [17, 18], with highest estimates found for repeat measurements that account for transient fluctuations [19]. Other studies have also probed the neural correlates of cognitive processes in the context of heritability using HCP data [20–23]. For instance, one study used bivariate genetic analyses to identify brain networks that were genetically correlated with cognitive tasks in math and language [21]. Similarly, another study found common genetic influences for white matter microstructure and processing speed [22]. Both studies demonstrated that heritability can provide a powerful link between brain and behavior.

Behavioral heritability is defined as the genetic contribution to the total variance for a phenotypic trait in a population, an important statistic for understanding individual differences. Twins (both monozygotic/MZ and dizygotic/DZ) are particularly useful for the estimation of heritability as they can help to differentiate the contribution of genes versus environment. In the classical twin design, the total phenotypic variance of a trait can be decomposed into four components: additive genetic influences (A, theoretically correlates at 1.0 for MZ twins and at 0.5 for DZ twins), non-additive genetic influences (D, theoretically correlates at 1.0 for MZ twins and at 0.25 for DZ twins), common environmental influences (C, theoretically correlates at 1.0 for both MZ twins and DZ twins) and unique environmental factors (E, theoretically uncorrelated for both types of twins). Due to modeling constraints, only three of these four parameters can be assessed simultaneously. When the MZ correlation is less than twice that of DZ correlation, non-additive genetic influences (D) are assumed to be negligible, resulting in the ACE model. When the MZ correlation is more than twice that of DZ correlation, common environmental influences are assumed to be negligible, resulting in the ADE model [6, 24–27]. Modern maximum likelihood-based modeling estimates various components for the total variance [28, 29], but in essence relies on the same set of assumptions and logic, which continue to be debated and depend on prior assumptions about the dataset. For example, researchers have different opinions on whether the equal environment assumption (EEA) is tenable. On the one hand, some believe that a greater level of physical similarity shared by MZ twins leads to more similar social environment. A separate argument is for the prenatal environment, MZ twins, especially monochorionic twins, are believed to share a more similar womb environment which is hypothesized to be important for certain developmental outcomes [30–34]. On the other hand, it has been argued that EEA is largely tenable as long as there is only a modest bias and the model is limited to few traits. One study controlling for environmental similarity between MZ and DZ pairs found significantly reduced heritability for only one out of 32 outcomes examined [35]. Another study concluded that the effects of chorionicity are small and limited to few phenotypes of the whole set of 66 phenotypes examined [36]. Furthermore, gene-environment interaction is often not properly modeled or completely omitted as in the case of using Falconer’s formula in twin studies [37]. Yet a recent meta-analysis paper that investigated the heritability of a wide range of human traits based on twin studies in the past fifty years showed that for 69% of the traits analyzed, there was a twofold difference in the MZ correlations relative to DZ correlations, consistent with a simple model that all twin resemblance was solely due to additive genetic variation [6]. Falconer’s formula thus continues to be used, but modifications to it, and assumptions behind it, depend on theoretical assumptions that may or may not be valid, depending on the details of the dataset.

Unlike traditional approaches in twin studies where researchers impose strong theoretical constraints on the exact causes for the difference between MZ and DZ twins, machine-learning tools have recently emerged as a model-free approach to such questions. Rather than describing genotype-phenotype associations based on the statistics of the population (as classical approaches do), machine-learning approaches learn multivariate patterns across individuals, and aim to produce a model that is predictive, i.e., that generalizes to held-out cases (as assessed, e.g., with cross-validation). The approach is entirely data-driven, and can reveal useful individual differences. However, a main challenge with machine-learning models is feature selection and model complexity, both of which can promote models that overfit the data and that fail to generalize. Feature selection and regularization are thus important; as well as comparisons across more than one type of model.

Several modern machine learning models, including the ones we use in the present paper, have been shown to outperform classical approaches across a number of different datasets [38]. These models have yielded notable improvements in the prediction of human phenotypic traits using single-nucleotide polymorphism (SNP) data [39–43]. One review that evaluated Ridge regression (which is a model used in our study) lists several advantages over conventional genome-wide association methods: (1) substantially increased accuracy, especially for large sample sizes; (2) the regularization term in the Ridge regression allows flexible accounting of the linkage disequilibrium between SNPs; (3) more computationally efficient than repeated simple regressions [39]. Other models, such as Random Forest, a nonlinear machine learning model, have been used to predict coronary artery calcification using SNP data, achieving not only good prediction, but also reliably identifying best predictors across different datasets [43]. Feature weights have been further utilized in one study that trained support vector machines (SVM) to classify siblings versus unrelated people using resting-state fMRI data to derive heritability for brain activity [44]. Overall, machine learning models have demonstrated superior prediction performance compared to conventional methods, and the feature weights learned by the models have the potential to be used for qualitative estimation of heritability (although feature weights need to be interpreted with caution (see Discussion; [45, 46]).

The present study had two broad aims: 1, We tried to re-identify the same individual (“fingerprinting”), and also identify an individual’s identical twin, based on their behavioral profile. We tested if the success in connectome fingerprinting that has been applied to the neuroimaging component of the HCP [3] could be replicated also using this set of rich behavioral measures. 2, We set out to characterize the relative heritability of the behavioral data in this dataset using the classical method, and at the same time using machine-learning models to classify MZ and DZ twins (from both raw behavioral scores and latent factors). Aside from valuable comprehensive data that describes the heritability of psychological variables in the HCP, our results motivate hypotheses about the heritability of the neural underpinnings, which we hope future studies will pursue in the same subject sample.

## Materials and methods

### Data

**Fig 1:**
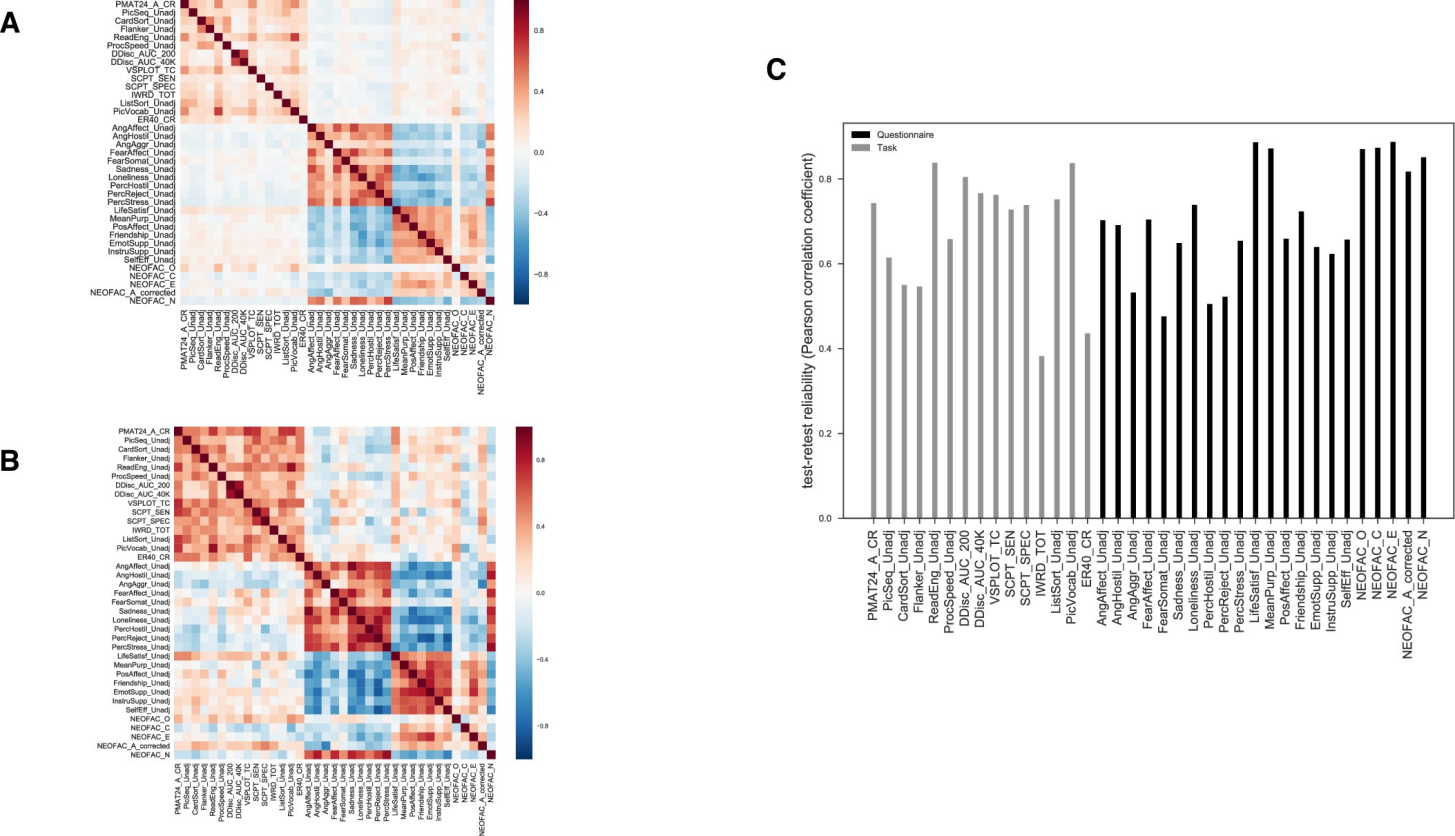
Overview of the dataset. (A) empirical correlation matrix for 37 behavioral variables in HCP (sample size N = 1189), color coded for Pearson’s correlation coefficient, (B) genetic correlation matrix for 37 behavioral variables, color coded for the magnitude of the bivariate genetic correlation (see text), (C) empirical test-retest reliability for 37 measures (sample size N = 46), color coded for domain. See S1 Table for descriptions of the variables.

We used behavioral data from the Human Connectome Project (HCP) S1200 release under the domains of cognition, emotion and personality [1]. The 37 selected variables were summary scores for either a behavioral task or a questionnaire (see S1 Table for more detailed description for each variable, and Fig 1A for their phenotypic correlation structure and Fig 1B for the genetic correlations). The NEO agreeableness score was re-calculated since item #59 was incorrectly coded at the time of downloading the data (an issue reported to and verified by HCP: https://www.mail-archive.com/hcp-users@humanconnectome.org/msg06007.html). Since the variables were on different scales, we first pre-processed them to all have zero mean and unit variance. Each subject was thus essentially described by a vector of 37 z-scored scores/features, representing their psychological profile.

Of 1206 subjects, 1189 subjects had complete data for the 37 scores of interest, and 1142 had family relationship data verified by genotyping, yielding a final set of 149 pairs of genetically confirmed monozygotic (MZ) twins (298 subjects, all of the same sex) and 90 pairs of dizygotic (DZ) twins (180 subjects, one twin pair was of opposite sex and thus excluded) with complete data for the 37 behavioral variables of interest. A subset of 46 MZ subjects had complete test-retest data for the selected 37 scores, which we used to calculate test-retest reliability (as their Pearson’s correlation coefficients, Fig 1C). We thus used 1189 subjects in total, of which 478 were either MZ or DZ twins.

### Bivariate genetic correlation

To estimate the degree of shared genetic influences among behavioral measures, we calculated bivariate genetic correlations using the SOLAR software package (https://www.nitrc.org/projects/se_linux). Briefly the total phenotypic correlation *ρ_P_* is partitioned into a genetic component *ρ_G_* and an environmental component *ρ_E_* according to the following model:

$$\rho_{P}=\sqrt{h_{a}^{2}{*h}_{b}^{2}}*\rho_{G}+\sqrt{\left(1-h_{a}^{2})*(1-h_{b}^{2}\right)}*\rho_{E}$$ (1)

where $h_{a}^{2}$ and $h_{b}^{2}$ correspond to the heritability for trait a and b, respectively. For all analyses, age, sex, age×sex, age^2^, age^2^×sex were included as covariates. Inverse Gaussian transformation was applied to ensure the normality of the measures.

### Same individual and twin identification

#### Same individual

We first asked how well a subject could be re-identified from their retest, compared to all other subjects, for the 46 subjects who had test-retest data available. We calculated pairwise Euclidean distances between a given subject’s retest data and each of the 1189 subjects’ original data (including the subject’s own original data) and then ranked the distances in ascending order to see if the subject’s retest data was closer to his/her own original data than the data from any other of the 1188 subjects.

#### MZ twin

Similar to the above, we took one person (target) out of the 298 MZ twins and calculated pairwise Euclidean distances between this subject and each of the remaining 1188 subjects, and then ranked the distances in ascending order to see if the corresponding MZ twin was closer to the target subject than were any of the other 1187 subjects.

In both cases, the Euclidean distance between two subjects was calculated as the following:

$$d\;(p,q)=\sqrt{\sum_{i=1}^{37}{\left(p_{i}-q_{i}\right)}^{2}}$$ (2)

Where **p, q** correspond to the 37-length vector representations for two subjects, and p_i_, q_i_ correspond to each of the individual behavioral measures.

#### Standard calculation of heritability

In the behavioral genetics literature, a standard way to derive heritability for the ACE model (contribution from additive genetic influences only) is based on twin correlations calculated using Falconer’s formula [25]:

$$h^{2}=2*(r_{MZ}-r_{DZ})$$ (3)

Where h^2^ is the overall heritability, r_MZ_ the correlation for a phenotypic trait between monozygotic twins, and r_DZ_ the correlation for a phenotypic trait between dizygotic twins.

Different a priori assumptions can modify this theoretical model. For example, if the ADE model is suggested, then r_MZ_ is taken as approximation for heritability which reflects the contributions from both the additive and non-additive genetic influences.

### Machine learning approach

We took as input data the absolute z-scored feature-wise difference between each twin and their co-twin, each described by a vector of 37 pre-processed behavioral variables as described above, giving us 149 MZ pair data and 90 DZ pair data which we tried to classify. Given that the MZ and DZ classes were not balanced (had different numbers of subjects), we randomly over-sampled the DZ class with replacement to match the number of individuals in the MZ class. This simple and conservative approach would prevent the models from focusing on learning the characteristics of the MZ class only, neglecting the examples from the DZ class, a common problem when machine-learning is used on unbalanced classes.

We used three widely used models: a Ridge classifier, a simple univariate model, and a Random Forest model, which is a nonlinear decision tree-based model that ensures accurate feature weights even when features are correlated. For the univariate model, the dependent variable was the class (MZ or DZ) and the independent variable was each of the 37 features; we used this simple model because it most clearly tests the maximal contribution of each feature in isolation. All three models were implemented in Python (v2.7.6) using the Scikit-learn (v0.20.3) library; the exact models used were sklearn.linear_model.LinearRegression, sklearn.linear_model.RidgeClassifier and sklearn.ensemble.RandomForestClassifier.

We fitted both Ridge (the alpha parameter for the regularization term was set to be alpha = 100 for using 37 features, alpha = 10 for using the set of 9 factor scores calculated using linear regression, and alpha = 100 for using both sets of 18 factor scores) and Random Forest models (maximum tree depth was set to be 5 with 100 trees in the forest to prevent overfitting). Each model was estimated 1000 times; for each iteration, data was sampled as described above and then randomly split into 70% training data and 30% testing data.

More specifically, the alpha parameter in the Ridge regression model, which reflects the regularization strength, was determined by the built-in cross-validation in the Scikit-learn library. We began with a wide range of initial alpha values (200,100,10, 1, 0.1, 0.01, 0.001) and the model estimated the best alpha value independently for 1000 iterations. We then re-ran the whole analysis using the single final alpha value that was chosen by the majority of the iterations to be the best regularization parameter.

For the Random Forest model, there is a larger set of hyper-parameters to consider. Given that we only had 239 unique data points available to learn to classify two classes, a thorough grid search for hyper-parameter optimization was not feasible and prone to overfitting. We therefore focused on three hyper-parameters that would most strongly affect model performance given prior knowledge (and fixed all other parameters to their default value), which were: number of estimators/trees, maximum depth of the decision trees and maximum number of features to consider for best split at each node. The architecture of the Random Forest model allowed us to make use of the out-of-bag samples that came for free as a validation set because for each tree, a bootstrap sample of the original training data with replacement was used and certain data points were left out. The generalization accuracy of the model was evaluated using the out-of-bag accuracy. More specifically, to determine the best number of trees, we tried out a list of values from 20 to 160, and at each given value, we ran three Random Forest models (maximum number of features to consider set to be ‘auto’, ‘log2’ and ‘none’ respectively) for 100 iterations to derive a stable mean out-of-bag accuracy. The result is shown in S1A Fig. We found that (a) setting maximum feature to be “none” performed considerably worse than the other two options, and (b) the out-of-bag accuracy increased first with more decision trees and then reached a plateau at around 100 trees. Therefore, the number of estimators was set to be 100 because setting it even higher did not improve model accuracy significantly and would be more computationally expensive. Similarly, to determine the optimal maximum depth, we tried out a list of values from 1 to 10 and also ‘none’. From S1B Fig, we observed again that setting the maximum feature to ‘none’ was considerably worse than the other two options which didn’t differ much from each other, and the out-of-bag accuracy increased first with more depth and then reached a plateau at around 5. Therefore, the maximum depth was set to be 5 and the maximum number of features to consider was set to be ‘auto’ which was the default option.

Ridge classification produced a measure of testing accuracy as well as the coefficients (weights) for each of the 37 behavioral features. For both Ridge and univariate models, we reported the absolute values for the coefficients. Detailed interpretation for the theoretical meaning of the sign of the coefficients is provided in the Discussion section. The Random Forest model returned feature importances (which are always positive and reflect mean decrease impurity (averaged across all decision trees in the random forest) [47]). So, a feature with a higher importance score is better at decreasing node impurity (which is a metric of the number of mis-labeled data points at the current node of a decision tree), i.e., it is more informative than other features. We evaluated the performance of Random Forest models using both testing accuracy and ROC curve analysis.

### Factor analysis

Given the strong inter-correlations between the 37 behavioral variables (Fig 1A) and the consideration that a single individual variable/task may yield an imprecise measure of the underlying psychological construct, we performed an exploratory factor analysis using SPSS with principal axis factoring as the extraction method, and kept nine factors that had eigenvalues >1, which together explained about 60% of the variance. This is not the only criterion for factor retention, therefore we included the scree plot in the supplement for readers’ own judgements. Factors were rotated using Promax rotation, since there was no evidence that the factors were orthogonal. We then calculated the factor scores using both regression and Bartlett methods. These factor scores were the features used for subsequent machine-learning prediction of heritability.

### Statistical testing

The statistical significance of our identification tests was evaluated with permutation testing. Over 1000 iterations, subject identity was randomly shuffled from the original dataset across the 1189 subjects, and the same identification procedures described above (both same-individual identification and identical-twin identification) were performed to derive the empirical distribution for chance-level identification accuracy.

To assess the statistical significance of our classification performance, we constructed the 95% confidence interval from the empirical distribution of the model’s prediction accuracy (resulting from the 1,000 bootstraps that we performed) for each classification problem. A bootstrap p-value was also computed as the ratio of the instances of having a testing accuracy equal to or lower than 50% (which is the expected chance accuracy for random guessing with equal probability for a balanced binary classification) out of the total number of bootstraps.

Permutation testing was also used to test for a significant difference in average heritability between the questionnaire domain and the behavioral task domain. The null hypothesis was that the task and the questionnaire domain comprised the same distribution. Under the null hypothesis, the number of all possible permutations (selecting 15 out of 37 measures as task scores) was 9.4*10^9^, which we approximated using Monte Carlo sampling of 100,000 permutations. For each permutation, we randomly assigned 15 values to the task domain and the rest to the questionnaire domain and then calculated the absolute difference between the two heritability means as our test statistic. Statistical significance was quantified as the probability (under the null hypothesis) of observing a value of the test statistic more extreme than what was actually observed. We performed the same analysis for four sets of estimates (standard heritability estimates, univariate model weights, Ridge weights, and feature importances for the Random Forest model, each consisting of 37 values).

## Results

### Individual re-identification, and monozygotic twin identification

A number of recent studies have attempted to identify individuals using different brain fingerprints, including functional connectivity [3, 4], structural connectivity [48], and white matter fiber geometry [49], motivated by the potential use of neural markers for precision medicine and precision psychiatry. Those studies have demonstrated that brain fingerprints can predict individual identity and behavioral outcomes. For example, using HCP data from 126 subjects, the whole brain connectivity matrix approach can identify individuals across two resting state scans with a success rate as high as 94% as well as predict intelligence score [3]. Given the rich set of behavioral measures that we have, we tested how well behavioral fingerprinting could work which would allow us to assess how reliable, robust and unique the behavioral profiles are for each individual.

We first attempted to re-identify the same individual using all of the 37 measures. Of the 46 subjects with retest data, we were able to re-identify 26 (meaning that the original data of those 26 individuals were closest to their retest data compared to all other 1188 subjects), yielding an accuracy of 56.5% (26/46) with a median distance rank of 1.0 and a mean distance rank of 12.1 among 1189 people. This suggests that even for cases where exact matches failed, the individuals were not that far from their own retest data (ranked on average as the 12th closest to their own retest data among 1189 subjects). We performed permutation testing to assess the statistical significance of our identification accuracy. Across 1,000 iterations, the highest success rate ever achieved on any one of the 1,000 permutations was 2/46 (roughly 4.3% and considerably lower than our observed accuracy). Since none of the 1,000 iterations ever exceeded our observed accuracy, the overall p-value associated with obtaining at least 26 correct identifications was the minimum we could estimate, that is p<0.001.

We carried out the same analysis for MZ twin identification: compared to other siblings and genetically unrelated people, MZ twins should be most similar to one another [24–26]. Of the 298 MZ subjects, we identified the exact corresponding MZ twin for 21 of them (i.e., the corresponding twins of those 21 individuals had the smallest distances to them when described by this set of behavioral measures compared to all other 1187 subjects), yielding an accuracy of 7.0% (21/298) with a median distance rank of 47.5 among 1188 people. This suggests that even for cases where exact matches failed, the MZ twins were not that far from each other (ranked at furthest as the 48th closest among 1188 subjects for half of the cases). Assessing statistical significance with 1,000 permutations, the highest success rate ever achieved on any one of the 1,000 permutations was 3/298, roughly 1.0%, and the p-value associated with obtaining at least 21 correct identifications was again the lowest possible, that is p<0.001. Thus, both our ability to identify somebody’s identical twin based on the behavioral data, and to re-identify the same individual, was highly statistically significant, even though accuracy for the first was considerably worse than for the second.

The ability to re-identify a given individual (that is, test-retest reliability) essentially sets an upper bound on the ability to identify a MZ twin, and presumably reflects the specific measurement limitations of this particular dataset, including factors such as the number of features (37 compared to ideally infinite) and the reliability of the features (test-retest reliability or measurement error in Fig 1C). However, both identifications (same individual and MZ twins) had the same set of limitations inherent to this dataset, and both groups share 100% genetic similarity. The lower prediction accuracy for MZ twins as compared to re-identification of the same individual thus suggests that the behavioral measures in our dataset have only low-moderate heritability, and that environmental factors may explain some of the individual differences in MZ twins (which is consistent with the literature as reviewed in the introduction). We next investigated the heritability of each measure and the fundamental assumptions of twin studies.

### The standard method of calculating heritability

**Fig 2:**
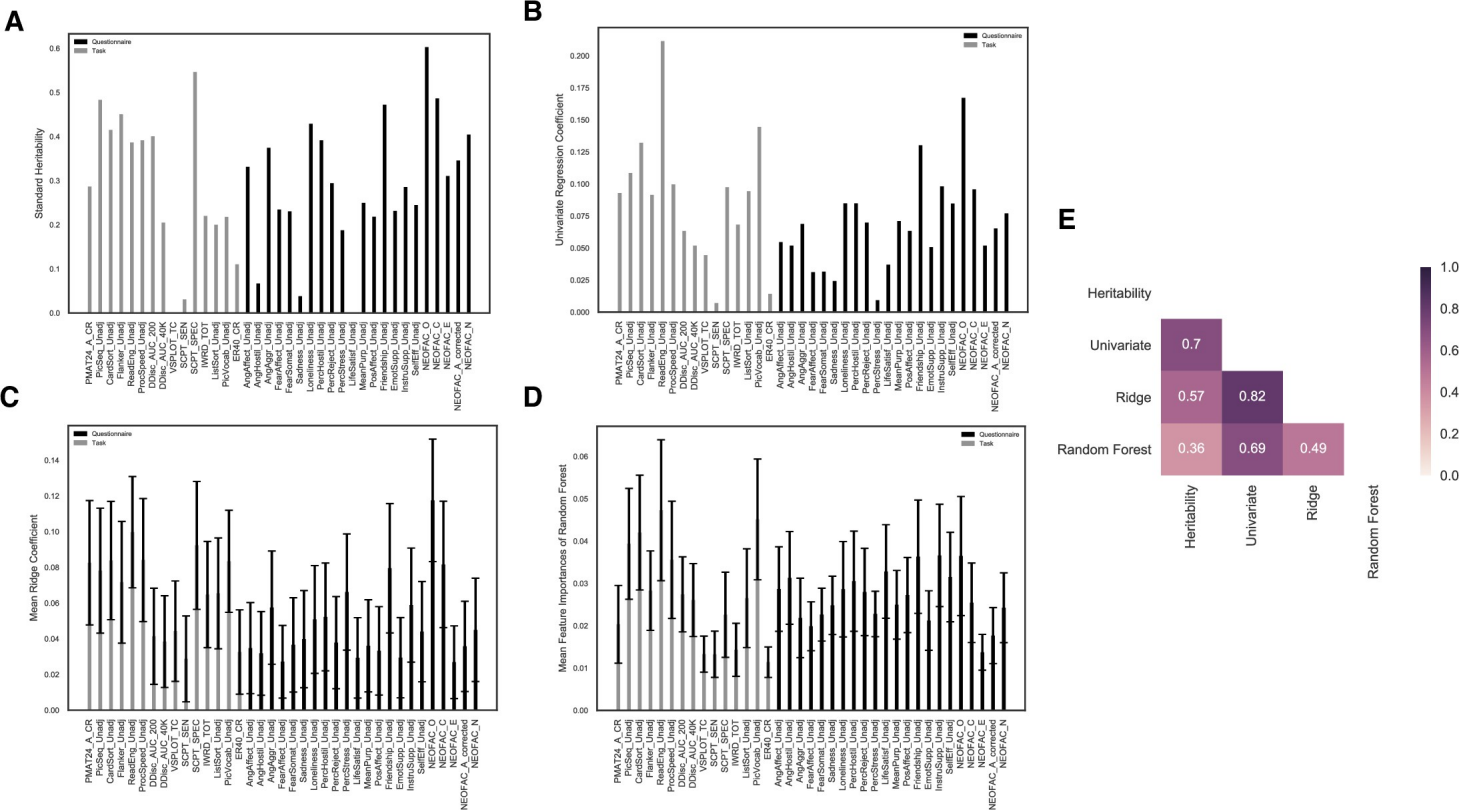
Heritability estimation across four methods for 37 behavioral measures. (A) standard heritability estimates; (B) univariate coefficients for each feature; (C) mean feature coefficients averaged across 1000 iterations for Ridge classifier (error bars represent standard deviation of coefficients); (D) mean feature importances averaged across 1000 iterations for Random Forest (error bars represent standard deviation of importances); (E) correlation matrix for four sets of heritability estimates assigned to 37 measures, color coded for Spearman’s rank correlation. See inset legend for details.

In twin studies, one of the most common approaches to calculate heritability is based on the difference in correlations between MZ and DZ twins (see Introduction). As can be seen from Fig 2A (and S1 Table), the heritability calculated in this manner had a large range across different tasks and surveys. For about half of the measures, the MZ correlation was less than two times the DZ correlation, suggesting the ACE model. On the other hand, for the other half of the measures, the MZ correlation was more than two times the DZ correlation, suggesting non-additive genetic contributions (ADE model). There were two measures whose MZ correlation was smaller than the DZ correlation, indicating no genetic contributions at all. One possible explanation for this result could be that the measures have poor test-retest reliability. Based on retest data from 46 subjects, the short Penn line orientation test had a test-retest reliability of 0.76 and the life satisfaction questionnaire had a test-retest reliability of 0.89. Another limiting factor could be the sample size used to calculate the twin correlations (on the order of 100 here and DZ twins were fewer than MZ twins). There exist more complex modeling approaches to estimate heritability [28, 29], but fundamentally, those methods rely on the same assumptions. The standard correlation-based calculations were simple and straightforward, but with many assumptions built in [27, 30–33, 37]. In this study, we explore the possibility of utilizing machine learning models that are more data-driven and less model-based to provide heritability estimates against which the standard heritability estimates could be compared.

### A machine learning approach to classify MZ and DZ twins

The traditional approach derives heritability from the differences between MZ and DZ twins at the population level. If we assume that any differences between the two types of twin pairs indeed arise solely from genetics, then in an ideal case with no noise components and no correlation among features, a classifier trained to distinguish MZ twins and DZ twins should assign greater weights to those features that have higher heritability, as they are more informative for discriminating the two classes. This allows us to test at least qualitatively how reasonable the heritability estimations were that we derived above using standard methods (see Discussion for some caveats in interpreting feature weights in multivariate linear regression).

The first approach we used was Ridge classification, which is a variant of a simple multivariate model with a regularization term that forces the weights to be more stable and robust to correlated features [50, 51] (which we had, as illustrated in Fig 1A). The mean coefficients for each feature are plotted in Fig 2C, the model had a mean testing accuracy of 68.7% (95% confidence interval for the testing accuracy: [58.9%,77.8%]; the bootstrap p-value under the null hypothesis that testing accuracy is not significantly higher than 50% was <0.001). In addition to Ridge regression, we also fitted the simplest univariate model for each of the 37 measures, an OLS regression model with a single feature, each one of the coefficients are shown in Fig 2B. This univariate regression would therefore reflect the maximal contribution from each feature in isolation, allowing a clearer quantification of each individual feature’s importance than the Ridge or Random Forest models, which incorporate multicollinearity between features. The two sets of coefficients (univariate and Ridge) had a Spearman’s rank-order correlation of 0.82 across the 37 features.

Another popular approach is the Random Forest classifier, which is a nonlinear model comprised of many decision trees. For each decision tree inside the forest, the method draws a randomly sampled training set and only considers a random sample of features for splitting at each node. The structure of the model helps with the problem of highly correlated features and allows more stable and accurate estimations of feature weights (importances). The mean feature importances are plotted in Fig 2D, the model had a mean predictive accuracy of 79.4% (95% confidence interval: [71.1%,87.8%]; p <0.001); mean area under the ROC curve was 0.88 (with a standard deviation of 0.04).

To compare all these different results, we quantified the correlations between all four sets of values, including classic heritability estimates, Ridge classifier coefficients, univariate model coefficients and Random Forest feature importances. We found good agreement across different approaches with Spearman’s rank correlation ranging from 0.36 to 0.82 (Fig 2E), which was consistent with our hypothesis that the heritability of a measure should be positively related to its contribution to the classification of MZ vs DZ twins. Considering that we had correlated features in the dataset (Fig 1A), the results also partially confirmed the capability of both Ridge and Random Forest at handling feature correlations as they both agreed well with the univariate coefficients, correlated at 0.82 and 0.69 respectively. Results that corrected for test-retest reliability were similar to the uncorrected ones presented here (S2 Fig).

We next asked a more general question: are the heritability or feature weights on average significantly different for the behavioral task domain compared to the self-report questionnaire domain? Significant domain-wise differences in terms of heritability could imply that these two domains have distinct genetic origins (and possibly distinct neural substrates as well) which would be of theoretical interest to researchers working on those measures [52]. The comparison was also motivated by the possibility of identifying one domain with significantly lower heritabilities, as that might suggest that environmental factors play a greater role for that domain and the possibility of developing more directed behavioral intervention for clinical populations.

**Fig 3:**
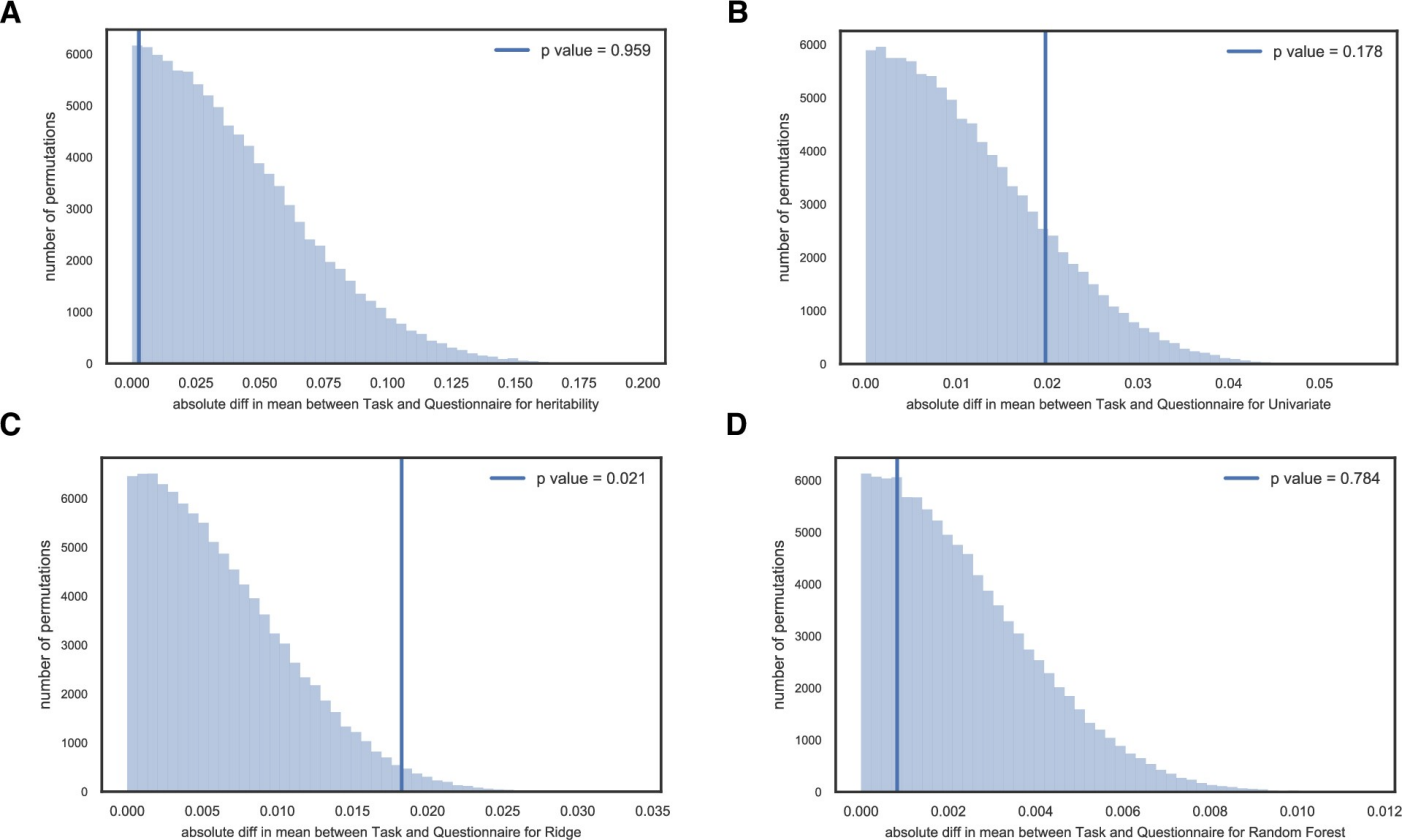
Distribution of the absolute mean difference between the task and questionnaire domain (vertical line indicates actual observation of the difference for average heritability between the task and questionnaire domain) for (A) standard heritability estimates; (B) univariate coefficients for each feature; (C) Ridge classifier coefficients; (D) Random Forest feature importances.

Under the null hypothesis that average heritability for the task and the questionnaire domain are not significantly different, we constructed the distribution of the absolute difference for average heritability between the task and questionnaire domain (Fig 3), and calculated the p-values for four sets of estimates (see more details in the Methods section). For all cases except Ridge classification (for which the p-value was 0.021, uncorrected for testing our hypothesis with the four sets of heritability estimates), we found no strong evidence to reject the null hypothesis. When taking test-retest reliability into consideration by simple disattenuation (dividing by rest-retest reliability), again only Ridge coefficients had the smallest p-value of 0.008 (S3 Fig). However, it may not be valid simply to divide by test-retest reliability, since measures with very poor reliability could yield artificially inflated heritability. It’s worth noting that the genetic correlation structure among measures (Fig 1B) did seem to imply that the two domains we tested here (tasks and questionnaires) have largely distinct genetic origins and perhaps distinct neural mechanisms as well, even though their heritabilities did not differ.

As noted before, there were strong inter-correlations among the 37 behavioral measures. We therefore used factor analysis to derive latent factors underlying those measures, an approach commonly taken when large numbers of tasks are available.

### Estimating heritability for the factors

As an example of this approach, we extracted nine factors from all 37 measures that together accounted for 59.7% of the total variance (S2 Table). Determining how many factors to retain, and the interpretations for those factors, were inevitably subjective. Retaining nine factors was more conservative (accounted for more variance) than what the scree plot would suggest (S4A Fig), which instead suggested three factors by visual inspection. In addition, we had a technical consideration that favored retaining a greater number of factors, since more features for classification models trained on latent factors would generally lead to better classification performance and thus more accurate classification of MZ/DZ twins. We offer the analysis of the heritability of latent factors as an exploratory complement to our main analysis of the heritability of the individual measures.

Our interpretations (which are subjective) and the accounted variances of the factors were as follows. Factor 1: positive social relationship (indicated by high positive loadings on measures such as emotional support and friendship survey) (22.2%); factor 2: negative affect (indicated by high positive loadings on measures such as fear, sadness and anger survey) (11.0%); factor 3: general intelligence (indicated by high positive loadings on a range of cognitive tasks with highest loading on the Penn Progressive Matrices test) (5.1%); factor 4: impulsivity (indicated by loadings on the two delay discounting tasks) (4.7%); factor 5: attention and processing speed (indicated by high positive loadings on the NIH card sort task, and the Flanker and Processing speed tests) (4.0%); factor 6: agreeableness (indicated by positive loading on NEO agreeableness and negative loading on anger aggression survey) (3.6%); factor 7: efficacy and conscientiousness (indicated by high positive loadings on NEO conscientiousness and self-efficacy survey) (3.2%): factor 8: language and communication (indicated by high positive loadings on two language tests and NEO openness) (3.2%) and factor 9: competitiveness (indicated by positive loadings on social distress and life satisfaction surveys) (2.8%).

**Fig 4:**
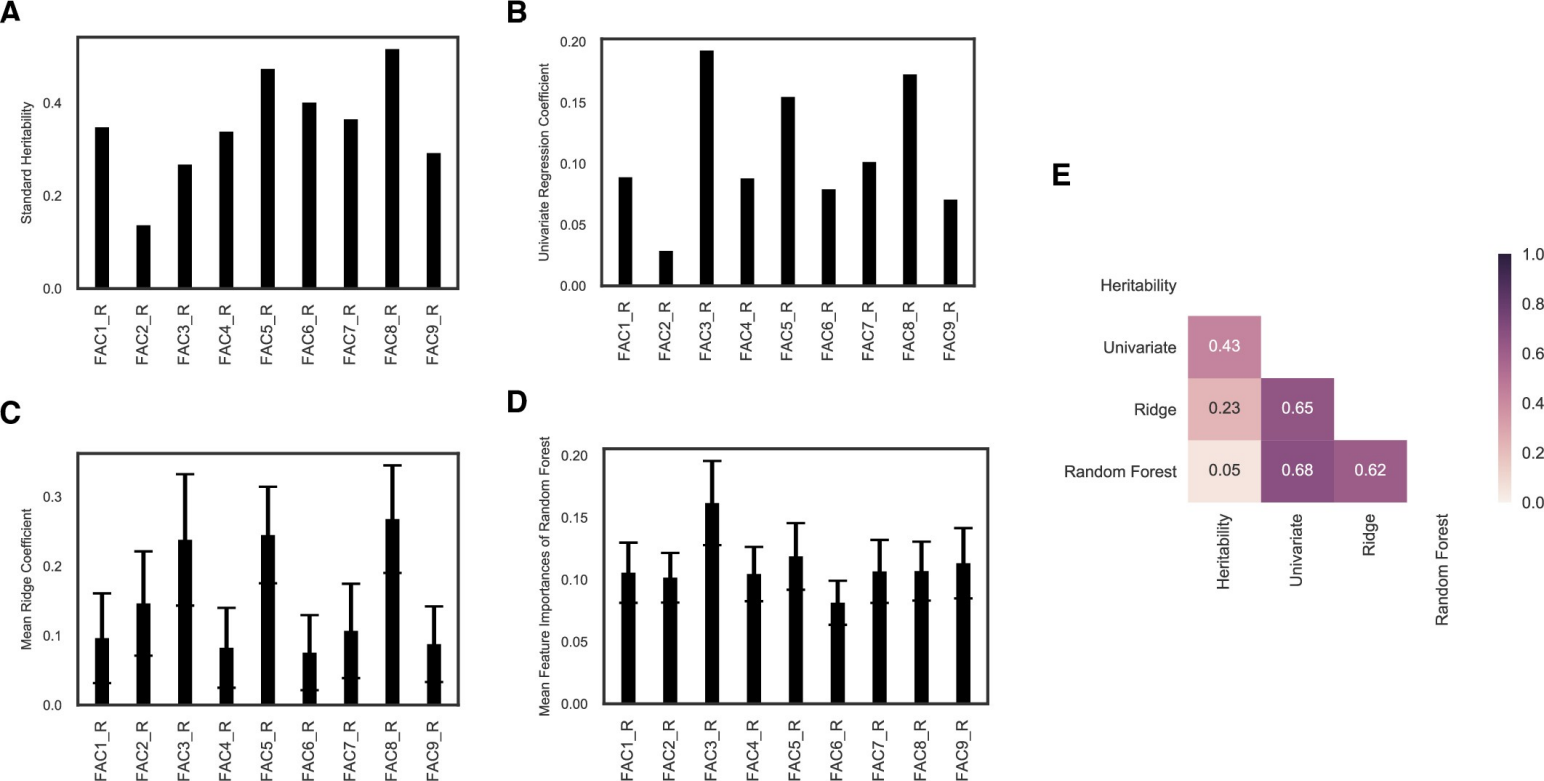
Heritability estimation across four methods for nine latent factors. (A) standard heritability estimates; (B) univariate coefficients for each factor; (C) mean feature coefficients averaged across 1000 iterations for Ridge classifier (error bars represent standard deviation of coefficients); (D) mean feature importances averaged across 1000 iterations for Random Forest (error bars represent standard deviation of importances); (E) correlation matrix for four sets of values assigned to 9 factors, color coded for Spearman’s rank correlation.

We repeated the previous analyses using the set of factor scores derived from regression methods so that each subject was represented by a vector of 9 factor scores to derive standard heritability, Ridge coefficients, univariate coefficients and Random Forest feature importances for the nine factors (Fig 4). When using the nine regression factor scores, The Ridge classifier had a mean accuracy of 64.2% (95% CI: [53.3%,73.3%]; bootstrap p-value = 0.006) while the Random Forest classifier had a mean testing accuracy of 77.9% (95% CI: [67.8%,86.7%]; bootstrap p-value <0.001) and mean area under the ROC curve of 0.86 (with a standard deviation of 0.04). The reduction of model performance compared to using all 37 measures was minimal, indicating that the latent factors captured the information relevant for the classification.

We also computed factor scores using both regression and Bartlett methods for reliability (since factor scores are indeterminate). These two methods produced two sets of very similar factor scores for the same nine factors (see correlation structure between all 18 factor scores in S4B Fig). We then used these two sets of factor scores simultaneously as features in the Ridge classifier and Random Forest model to further assess the ability of each model to handle highly correlated features (a more challenging task than handling the 37 variables which were less inter-correlated in comparison). For a model that’s robust to correlation among features, it should be able to assign similar weights or importances to features that are highly correlated with each other. For standard heritability and univariate coefficients (S5A and S5B Fig), each factor score was treated independently, so they were not susceptible to the influence of correlation among factors. For the Ridge classifier, for the two sets of factor scores, the two sets of coefficients (S5C Fig) had a Pearson’s correlation of 0.79. For the Random Forest analysis, the correlation between the two sets of feature importances (S5D Fig) was 0.61. Therefore, these results further confirmed that Ridge and Random Forest were able to assign similar weights to highly correlated features and that their estimation of feature weights was reliable. In addition, for the set of factor scores derived by regression, when trained alone versus together with the other set of factor scores computed by the Bartlett method, the Spearman’s rank correlation of Ridge coefficients was 0.73. For the Random Forest classifier, the feature importances were correlated at 0.93. These results demonstrated that the feature weights that Ridge and Random Forest learned for the nine factors (when factor scores were calculated using the Regression method) were robust and consistent.

We also carried out the same analysis using only the first three factors as the scree plot would suggest (S6 Fig). The Ridge classifier had a mean accuracy of 59.5% (95% CI: [48.9%,70.0%]; bootstrap p-value = 0.044) while the Random Forest classifier had a mean testing accuracy of 65.7% (95% CI: [55.6%,75.6%]; bootstrap p-value = 0.001). The general pattern of heritability estimation across methods was similar with the calculations using all nine factors but the rank correlations were of lower resolution given the smaller number of factors.

Recall that for the 37 original measures, standard heritability and feature importances from the three models agreed relatively well, from 0.36 to 0.7 (Fig 2E). However, for the nine factors, the classical heritability estimates (Fig 4A) had lower correlations with the three other sets of model estimation, from 0.05 to 0.43 (Fig 4E). One specific difference, for example, was the estimation of factor 3 which reflects general intelligence. All three models assigned high importance to this factor while the traditional heritability calculation assigned a rather low value at 26.6%. In the literature, the heritability of intelligence has been studied extensively, often utilizing twin studies. The heritability of intelligence generally increases with age, estimated to be about 20% in infancy to perhaps 80% in later adulthood. One possible explanation for this trend is that small genetic differences get magnified as children select, modify and create their own experiences on the basis of their genetic propensities (so called genetic amplification). The HCP subjects are all in the age range of 22–35 years (young adulthood) and the heritability of intelligence in this age range is estimated around 60%-80% [53–57]. The machine-learning models are thus likely to have produced a more qualitatively accurate estimation of heritability from this dataset than the standard approach was able to.

## Discussion

### Summary of results

The goal of our study was to characterize the heritability of behavioral measures in the Human Connectome Project (HCP), given the prominence of this dataset in investigations of brain-behavior relationships and individual differences. We analyzed a comprehensive set of 37 behavioral scores in the HCP. When representing each subject using this set of behavioral data, we were able to achieve a behavioral fingerprinting accuracy of 56.5% (re-identification of the same individual), and in the case of identifying identical twins, an accuracy of 7.0% (both very significantly above chance as determined with permutation tests). We further computed heritability for all 37 behavioral scores using both a classical correlation-based method as well as three machine-learning based methods (univariate linear model; Ridge classification model and Random Forest model) that were trained to distinguish MZ twins from DZ twins. We found relatively high correlations between the two schemes (Fig 2E). Given the inter-correlations among the 37 scores, an exploratory factor analysis was conducted to extract nine latent factors, whose heritability we assessed similarly. In this case, the correlations between the classical heritability estimation and machine-learning-based model weights were lower (Fig 4E).

### Same individual and MZ twin identification

Our behavioral fingerprinting scheme was inspired by the success of connectome fingerprinting (based on a neuroimaging-derived measure) using HCP data [3]. Our accuracy of 56.5% was relatively high considering the limiting factors that we faced: a small number of features compared to the connectome fingerprinting (where each subject was described as a 268*268 connectivity matrix which essentially gave 35778 unique neuroimaging-derived features) and measurement error from some measures with relatively low test-retest reliability. For example, in the cognitive task domain, the Penn word memory task (IWRD_TOT) may have had the lowest test-retest reliability in part because it targets verbal memory in a novel situation, which is a form of fluid ability that is less consistent across time. In contrast, cognitive tasks that target crystallized abilities, such as the oral reading recognition task (ReadEng_Unadj) and the picture vocabulary task (PicVocab_Unadj), had the highest reliabilities. For the social and affect domain measures in the form of questionnaires, we observed that the NEO personality dimensions had superior test-retest reliability, which is consistent with the relative temporal stability of personality traits. Some of the least reliable measures in this domain were questionnaires about state perception of negative affect in social settings (FearSomat_Unadj, PercHostil_Unadj, PercReject_Unadj). This was also expected, since the test and retest measures on these variables were obtained at different times, making it possible for the same individual to actually vary in the case of less temporally stable state variables such as these. It should also be noted that the statistical power of our behavioral fingerprinting was limited by the sample size of the HCP dataset where only 46 subjects had complete retest behavioral data. Ideally, a larger sample size and a richer set of psychological measures will provide a more accurate and reliable estimation for the behavioral fingerprinting accuracy.

Our identification of MZ twins faced the same limitations, but we observed a drop of performance to an accuracy of 7.0% (still highly significantly better than chance). This accuracy drop alone would seem to put a limit on the strength of the heritability of our measures. One possible explanation is that the unique environment actually accounts for a substantial portion of the variance for those measures, overwhelming the contribution of common environment and genes. According to a study that used maximum likelihood modeling, unique environment does account for the majority of variances for many of the measures in the HCP, including some of the ones we selected [29]. This may also partly explain the modest classification accuracy of Ridge classification between MZ twins and DZ twins, since stronger contribution of unique environment implies weaker contribution of genetics and common environment to the overall phenotypic variances, thus diminishing group differences between MZ twin pairs and DZ twin pairs.

### Comparison of the standard correlation-based heritability estimation versus machine-learning-based model weights

The standard analysis calculates the heritability based on the MZ and DZ correlations for a phenotypic trait. Depending on the relative ratio between the twin correlations, researchers choose to include either the common environment (C) or non-additive genetic influences (D) in the model. It should be noted that since those two factors compete towards making the MZ correlation more or less than two times the DZ correlation, if C and D both exist, they can mask the effects of one another [58]. In our calculations, we also found that two measures that had good test-retest reliability had lower MZ correlations compared to DZ correlations. Possible reasons for ‘negative heritability’ could be due to small sample size and/or lack of explicit knowledge of the common environment. However, it should be mentioned that a negative estimation of heritability is not rare using such methods and although most researchers attribute such invalid results to noise, they could in fact be evidence against the assumptions behind the calculations [37, 59].

In addition, we trained machine learning models to distinguish MZ twin pairs and DZ twin pairs and used the model weights to verify the validity of the traditional heritability estimates. Measures/features that have high heritability are expected to contribute more to the classification. We found good rank correlations between the standard heritability and another three sets of model coefficients for the 37 behavioral variables (Fig 2E). More specifically, for measures with good test-retest reliability such as the five NEO personality traits, we observed consistent estimation of heritability across all four methods both in terms of their magnitudes among all of the 37 measures and the magnitudes relative to one another. On the other hand, for some measures such as the Penn word memory task (IWRD_TOT) and the Penn emotion recognition task (ER40_CR), the poor test-retest reliability seemed to put a ceiling on the magnitude and reliability of the heritability estimation across methods. The openness personality dimension (NEOFAC_O) had the highest and most consistent heritability estimation across all four analytic methods in the questionnaire domain and the oral reading recognition task (ReadEng_Unadj) had the highest average heritability estimation in the task domain; heritability estimates for these two tasks were fairly consistent across models, but less consistent with the standard estimates.

When applied to nine latent factors, the agreements between the standard heritability estimate and the three sets of model coefficients were substantially lower (Fig 4E). However, the three machine learning models had good agreement with one another, as shown by relatively high rank correlations (all above 0.6) (Fig 4E). As mentioned above, the standard heritability estimation for the general intelligence factor deviated greatly from the other three models, and from the literature. Such disagreement raises concerns about the validity of the assumptions made by the classical twin study and the usage of traditional methods for calculating heritability.

### Limitations and future directions

To the best of our knowledge, this is the first comprehensive characterization of the heritability of the behavioral measures in the full HCP database, and the first application of utilizing machine learning models to address this question. We will evaluate each model respectively and make recommendations for future use.

For the univariate linear model, a conceptually simple model, each measure was evaluated independently for its maximal contribution for the classification. For both raw measures and latent factors, univariate model coefficients agreed best with standard heritability calculations. Though it should be noted that given the shortcomings of standard calculations that we discussed before, good agreement with these doesn’t necessarily imply agreement with the true set of heritability values.

The second model we used was a Ridge classifier, a commonly used linear model to deal with correlated features [50, 51, 60]. A recent paper (using single-nucleotide polymorphism data) concludes that Ridge classification will improve predictive accuracy substantially compared to standard repeated univariate regression for a large enough sample size [39]. As a regularized regression, Ridge has proven to be effective at handling feature correlation, illustrated by its good agreement with the univariate coefficients (Figs 2E and 4E) and its ability to assign similar weights to the two sets of factor scores (S5C Fig). However, it is important to note that, in general, multivariate linear models such as Ridge are better suited for decoding and prediction purposes, whereas the interpretation for feature weights in the presence of noise and feature correlations can be problematic. The reason is that multivariate linear classification will assign weights to features based on the overall (multivariate) performance, which means that feature weights are not independent. In an extreme case, a feature (behavioral measure) that by itself contains no relevant information for the classification may be assigned a large weight because it cancels out shared noise present in other features [45]. In our study, our confidence in interpreting the Ridge weights comes from two considerations: 1, the high agreement between the univariate weights (whose weights do not suffer from the same caveat) and the Ridge ones, correlated at 0.82 and 0.65 for the case of 37 measures and 9 factors, respectively. 2, after transforming the Ridge weights into activation patterns of the corresponding forward model (see [45] for details and mathematical formulas), we observed a similar level of agreement with the univariate model, correlated at 0.84 for the 37 measures. For these reasons, we believe that the Ridge weights, in our case, were largely reasonable to be interpreted as relative heritability strengths.

For both the univariate regression model and the Ridge classification model (which is essentially a Ridge regression model with an addition step of taking the sign of the regression outcome), MZ twins were labeled as +1 while DZ twins were labeled as -1 in our implementation. So, both models tried to predict more positive regression outcomes for MZ twins compared to DZ twins. Theoretically, given the expectation that MZ twins have smaller absolute discrepancy than DZ twins for each measure (reflected by smaller absolute difference in their z-scored behavior scores for each measure), the sign of the coefficients should be negative such that the linear combination of the features for MZ twins will be more positive than DZ twins. Furthermore, the absolute magnitudes of the coefficients from these two regression models should reflect the informativeness of each feature. Because the features are z-scored scores for each behavior measure, the same amount of change for a feature with a coefficient of larger absolute magnitude will result in a larger absolute shift for the regression outcome. In our analysis, we found most of the coefficients to be negative with occasional positive coefficients that were of small magnitude. Possible explanations for the unexpected positive coefficients include: unstable estimation due to small sample size of HCP (on the order of 100 per class, which is also modest for twin studies); random measurement errors and also possibly innate errors due to a lack of explicit knowledge on common environment (which means our assumption that MZ/DZ twins share the same common environment might be violated). For this study, we couldn’t rule out the possibilities listed above and therefore chose to report the absolute values of the coefficients conservatively instead of concluding that the positive coefficients indicate theoretically invalid results.

The Random Forest model was also robust with respect to correlations among features (e.g., S5D Fig, for two sets of almost identical factor scores for the same nine factors, the two sets of feature importances had a Pearson’s correlation of 0.61), and achieved the highest accuracy for the classification between MZ twin pairs and DZ twin pairs. It does not suffer from the caveat that we noted above for Ridge classification with respect to interpreting the feature weights. One reason for the superior performance of Random Forest compared to the other approaches is likely its ability to find nonlinear separation of the two classes, which is impossible for linear models, especially in the presence of noise. A second reason is the property of “bagging” in the model; specifically, for each tree, the training data is a bootstrap sample of the original training sample with replacement, meaning that each tree has slightly different training data. Also, for each node of a decision tree in the random forest, it only makes use of a subset of features, which further de-correlates the trees. So, by averaging the prediction of those shallow trees (maximum tree depth set to be 5 in our case), the end result would reduce variance and prevent the random forest from overfitting (unlike a single deep decision tree). Given the nonlinear nature of the model, though, the feature importances should be interpreted in a qualitative sense rather than in an absolute sense. In addition, because feature importance scores are always positive, it’s not possible for them to signal the sign of effect, as was the case with the regression coefficients.

In this study, we focused on the classification of MZ twins versus DZ twins as a starting point, because within the classical twin study framework, the model weights in this classification scheme have a clear theoretical interpretation (that they should only reflect contribution from genetics). Weights derived from classification of MZ twins versus genetically unrelated people, for example, would reflect a complex mixture of genetic effects and common environment, which would be difficult to interpret. However, future research could explicitly quantify the common environment (the HCP does not provide such information, besides household ID), and even propose new models to explain the composition of the total phenotypic variance. Researchers could then train multiple classifiers (such as MZ versus DZ, full siblings versus half siblings) to further disambiguate the contribution of each component.

This general machine-learning framework could be applied to the qualitative estimation of heritability of brain activation as well, a source of data much more mined in the HCP than the phenotypic data. One recent study organized a subset of HCP subjects into MZ twins, DZ twins, siblings and unrelated people and found greater activation pattern similarity with greater genetic relatedness [61]. Using our approach, such findings could go beyond simple association to heritability estimation, by training classifiers on brain activation patterns for different groups. In summary, the machine learning methods that we introduced here have the potential to not only supplement standard heritability calculations, but also to provide insights for theories explaining phenotypic variance, and studies that focus on linking brain activation with behavior.

## Supporting information

S1 Fig Hyper-parameter optimization for the Random Forest model. (A) the general trend of how the number of estimators (x-axis) affects the out-of-bag accuracy (y-axis) and (B) the general trend of how the maximum depth of the decision trees (x-axis) affects the out-of-bag accuracy (y-axis). Each point represents the mean out-of-bag accuracy across 100 iterations and three different conditions for maximum feature (‘auto’,’log2’and ‘none’) are color-coded as shown in the figure legend.
(TIF)

S2 Fig Spearman’s rank correlation matrix for four sets of heritability estimates assigned to 37 measures that are corrected for test-retest reliability. (TIF)

S3 Fig Distribution of the absolute mean difference between the task and questionnaire domain (vertical line indicates actual observation) for (A) standard heritability estimates; (B) univariate coefficients for each feature; (C) Ridge classifier coefficients; (D) Random Forest feature importances. All values are corrected for test-retest reliability.
(TIF)

S4 Fig (A) Scree plot for the factor analysis; (B) Pearson’s correlation matrix for two sets of factor scores derived using regression method (FAC1_R to FAC9_R) and Bartlett method (FAC1_B to FAC9_B).
(TIF)

S5 Fig Heritability estimates for latent factors. (A) standard heritability estimates; (B) univariate coefficients for each feature; (C) mean feature coefficients averaged across 1000 iterations for Ridge classifier (error bars represent standard deviation of coefficients); (D) mean feature importances averaged across 1000 iterations for Random Forest (error bars represent standard deviation of importances for two sets of factor scores (color coded for Regression and Bartlett).
(TIF)

S6 Fig Heritability estimation across four methods for the first three latent factors. (A) standard heritability estimates; (B) univariate coefficients for each factor; (C) mean feature coefficients averaged across 1000 iterations for Ridge classifier (error bars represent standard deviation of coefficients); (D) mean feature importances averaged across 1000 iterations for Random Forest (error bars represent standard deviation of importances); (E) correlation matrix for four sets of values assigned to 3 factors, color coded for Spearman’s rank correlation.
(TIF)

S1 Table List of 37 behavioral variables selected with basic descriptions, twin correlations and heritability estimates. (DOCX)

S2 Table Loading matrix for factor analysis. The Table shows the loadings of each of the 37 measures (rows) onto the 9 factors that we derived (absolute values less than 0.30 were suppressed).
(DOCX)

S3 Table List of 9 latent factors with interpretations, twin correlations and heritability estimates. (DOCX)
