# Supplementary figures and images for: Estimating the heritability of psychological measures in the Human Connectome Project dataset
Source: PLoS One. 2020 Jul 9;15(7):e0235860. doi: 10.1371/journal.pone.0235860 (PMC7347217; doi:10.1371/journal.pone.0235860)

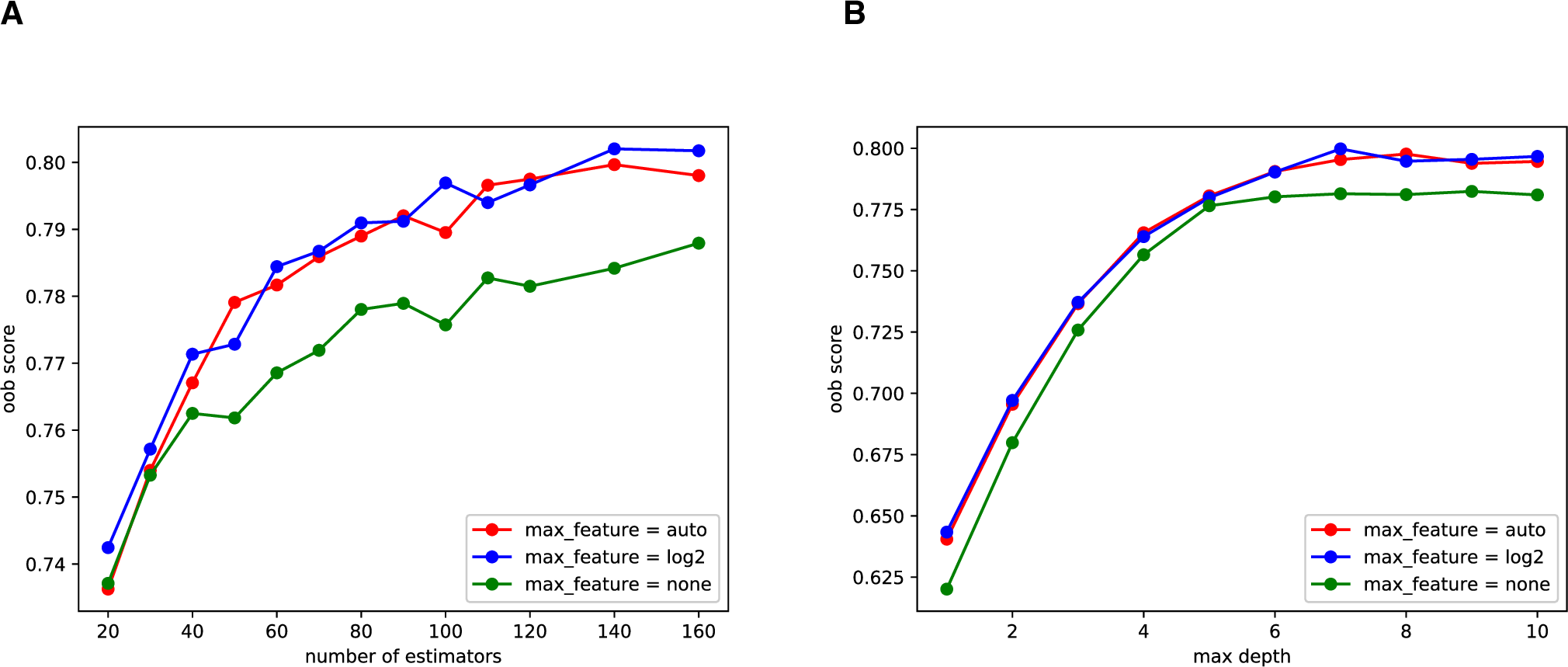

Supplement: S1 Fig — (A) the general trend of how the number of estimators (x-axis) affects the out-of-bag accuracy (y-axis) and (B) the general trend of how the maximum depth of the decision trees (x-axis) affects the out-of-bag accuracy (y-axis). Each point represents the mean out-of-bag accuracy across 100 iterations and three different conditions for maximum feature (‘auto’,’log2’and ‘none’) are color-coded as shown in the figure legend. (TIF) [file pone.0235860.s001.tif]

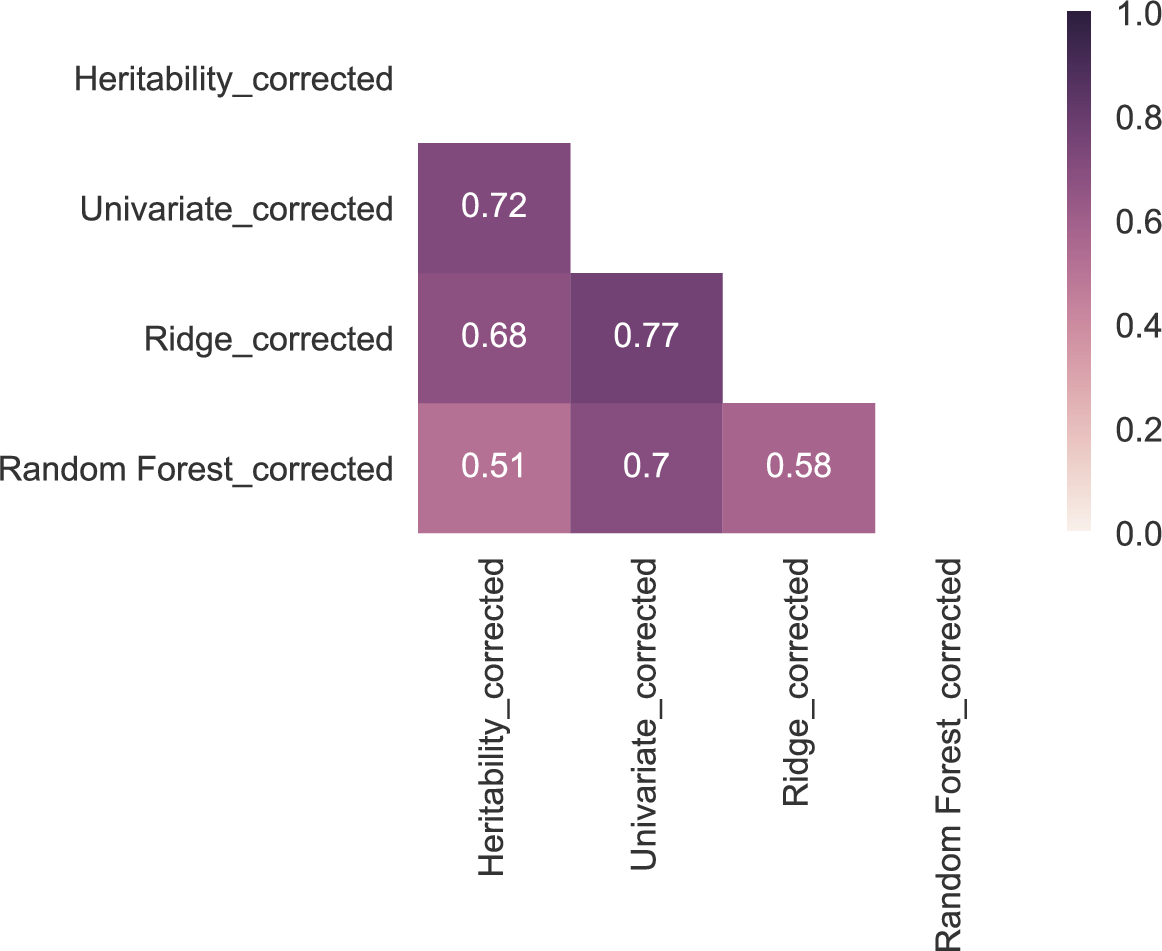

Supplement: S2 Fig — (TIF) [file pone.0235860.s002.tif]

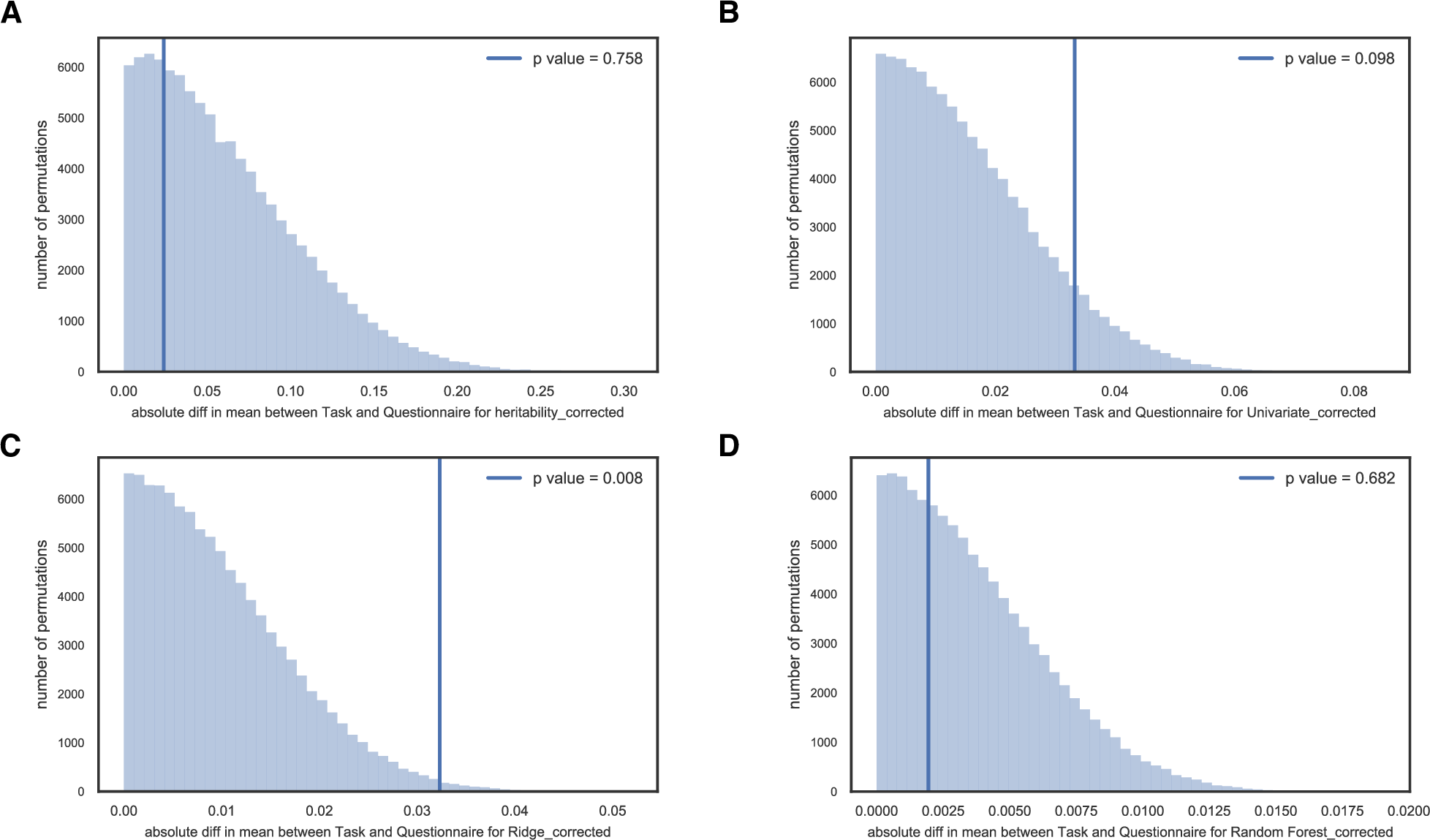

Supplement: S3 Fig — Distribution of the absolute mean difference between the task and questionnaire domain (vertical line indicates actual observation) for (A) standard heritability estimates; (B) univariate coefficients for each feature; (C) Ridge classifier coefficients; (D) Random Forest feature importances. All values are corrected for test-retest reliability. (TIF) [file pone.0235860.s003.tif]

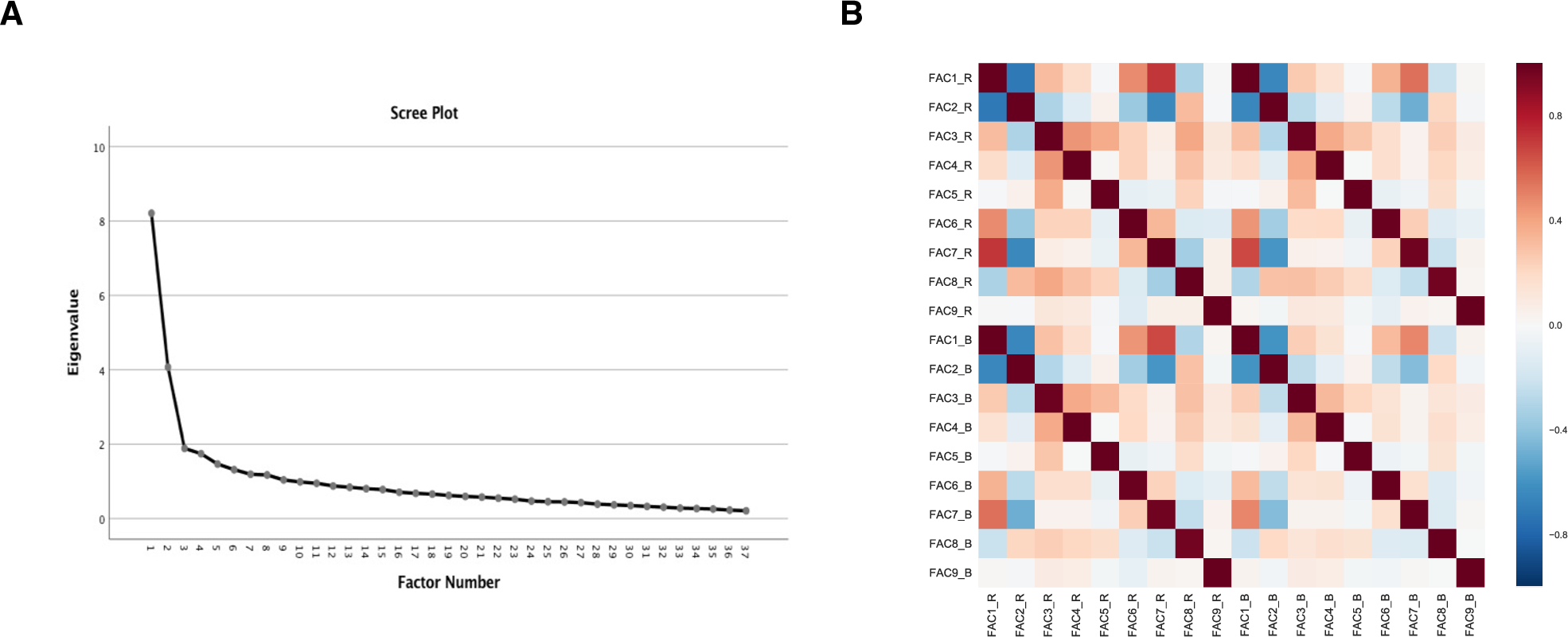

Supplement: S4 Fig — (A) Scree plot for the factor analysis; (B) Pearson’s correlation matrix for two sets of factor scores derived using regression method (FAC1_R to FAC9_R) and Bartlett method (FAC1_B to FAC9_B). (TIF) [file pone.0235860.s004.tif]

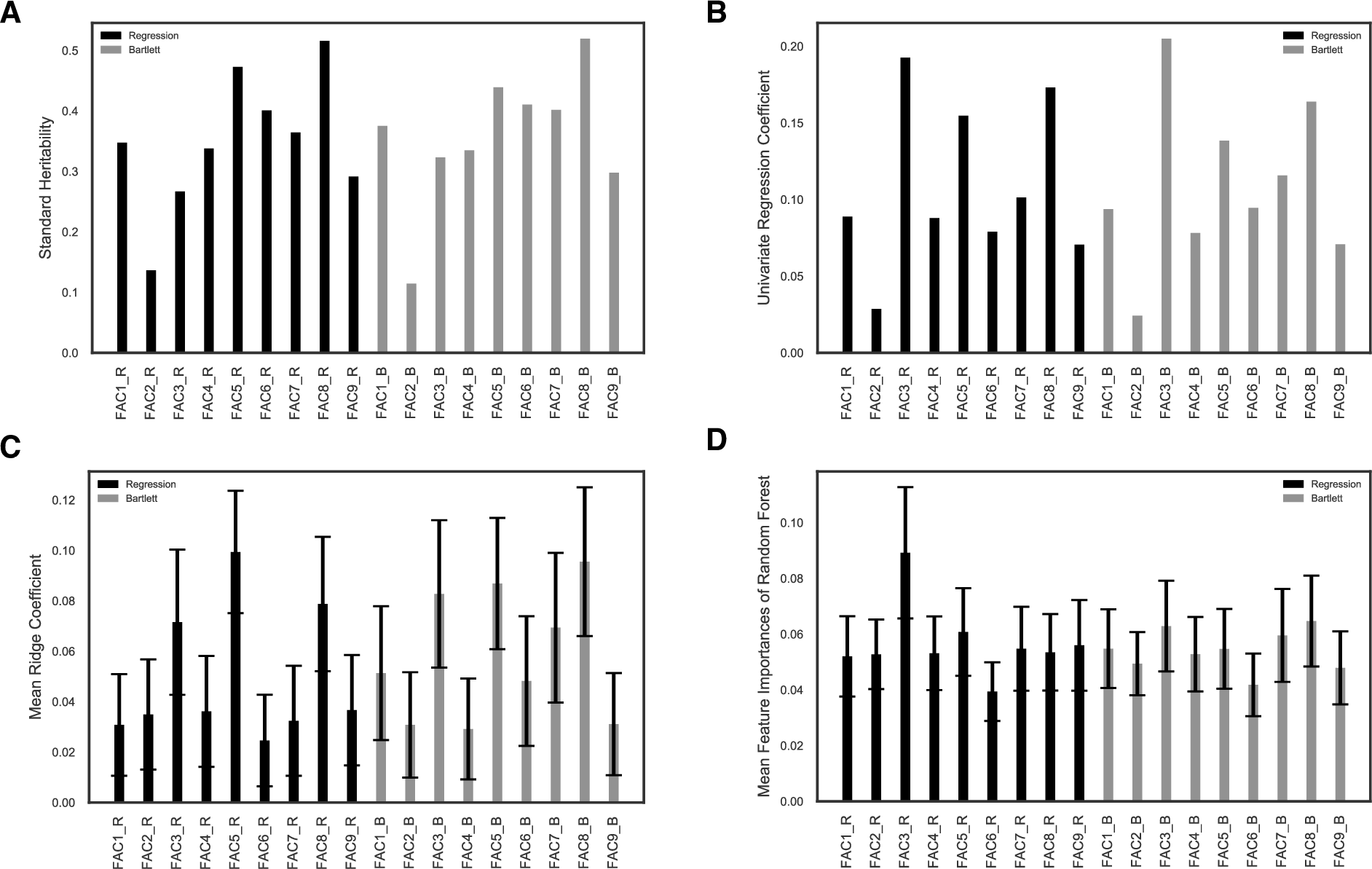

Supplement: S5 Fig — (A) standard heritability estimates; (B) univariate coefficients for each feature; (C) mean feature coefficients averaged across 1000 iterations for Ridge classifier (error bars represent standard deviation of coefficients); (D) mean feature importances averaged across 1000 iterations for Random Forest (error bars represent standard deviation of importances for two sets of factor scores (color coded for Regression and Bartlett). (TIF) [file pone.0235860.s005.tif]

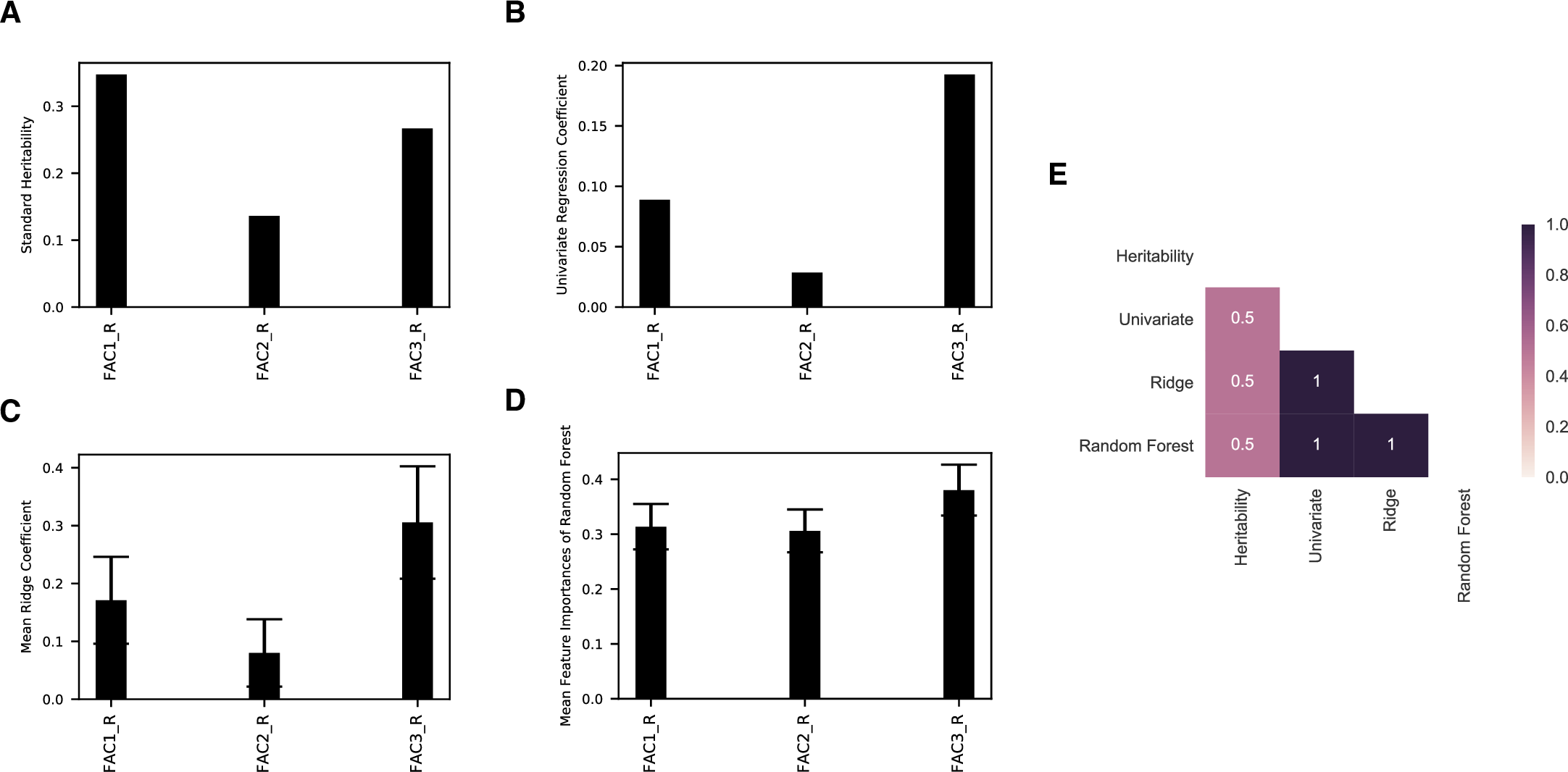

Supplement: S6 Fig — (A) standard heritability estimates; (B) univariate coefficients for each factor; (C) mean feature coefficients averaged across 1000 iterations for Ridge classifier (error bars represent standard deviation of coefficients); (D) mean feature importances averaged across 1000 iterations for Random Forest (error bars represent standard deviation of importances); (E) correlation matrix for four sets of values assigned to 3 factors, color coded for Spearman’s rank correlation. (TIF) [file pone.0235860.s006.tif]
