# Supplementary material for: Estimating the heritability of psychological measures in the Human Connectome Project dataset
Source: PLoS One. 2020 Jul 9;15(7):e0235860. doi: 10.1371/journal.pone.0235860 (PMC7347217; doi:10.1371/journal.pone.0235860)
Supplement: S1 Table — (DOCX) [file pone.0235860.s007.docx]

**S1_Table.** List of 37 behavioral variables selected with basic descriptions, twin correlations and heritability estimates.

| HCP variable name | Description | Task/ questionnaire | rMZ | rDZ | heritability |
| --- | --- | --- | --- | --- | --- |
| PMAT24_A_CR | Penn Progressive Matrices | Task | 0.513 | 0.369 | 0.288 |
| PicSeq_Unadj | NIH Toolbox Picture Sequence Memory Test | Task | 0.483 | 0.237 | 0.483 |
| CardSort_Unadj | NIH Toolbox Dimensional Change Card Sort Test | Task | 0.415 | 0.147 | 0.415 |
| Flanker_Unadj | NIH Toolbox Flanker Inhibitory Control and Attention Test | Task | 0.450 | 0.136 | 0.450 |
| ReadEng_Unadj | NIH Toolbox Oral Reading Recognition Test | Task | 0.732 | 0.539 | 0.386 |
| ProcSpeed_Unadj | NIH Toolbox Pattern Comparison Processing Speed Test | Task | 0.391 | 0.043 | 0.391 |
| DDisc_AUC_200 | Delay Discounting: $200 | Task | 0.400 | 0.182 | 0.400 |
| DDisc_AUC_40K | Delay Discounting: $40,000 | Task | 0.496 | 0.394 | 0.204 |
| VSPLOT_TC | Variable Short Penn Line Orientation | Task | 0.394 | 0.518 | 0^*^ |
| SCPT_SEN | Short Penn Continuous Performance Test: Sensitivity | Task | 0.031 | -0.065^#^ | 0.031 |
| SCPT_SPEC | Short Penn Continuous Performance Test: Specificity | Task | 0.546 | 0.213 | 0.546 |
| IWRD_TOT | Penn Word Memory Test | Task | 0.343 | 0.233 | 0.22 |
| ListSort_Unadj | NIH Toolbox List Sorting Working Memory Test | Task | 0.558 | 0.458 | 0.2 |
| PicVocab_Unadj | NIH Toolbox Picture Vocabulary Test | Task | 0.671 | 0.562 | 0.218 |
| ER40_CR | Penn Emotion Recognition Test | Task | 0.160 | 0.105 | 0.11 |
| AngAffect_Unadj | NIH Toolbox Anger-Affect Survey | Questionnaire | 0.331 | 0.093 | 0.331 |
| AngHostil_Unadj | NIH Toolbox Anger-Hostility Survey | Questionnaire | 0.331 | 0.298 | 0.066 |
| AngAggr_Unadj | NIH Toolbox Anger-Physical Aggression Survey | Questionnaire | 0.374 | 0.172 | 0.374 |
| FearAffect_Unadj | NIH Toolbox Fear-Affect Survey | Questionnaire | 0.373 | 0.256 | 0.234 |
| FearSomat_Unadj | NIH Toolbox Fear-Somatic Arousal Survey | Questionnaire | 0.230 | 0.059 | 0.230 |
| Sadness_Unadj | NIH Toolbox Sadness Survey | Questionnaire | 0.294 | 0.275 | 0.038 |
| Loneliness_Unadj | NIH Toolbox Loneliness Survey | Questionnaire | 0.429 | 0.106 | 0.429 |
| PercHostil_Unadj | NIH Toolbox Perceived Hostility Survey | Questionnaire | 0.391 | 0.064 | 0.391 |
| PercReject_Unadj | NIH Toolbox Perceived Rejection Survey | Questionnaire | 0.294 | 0.009 | 0.294 |
| PercStress_Unadj | NIH Toolbox Perceived Stress Survey | Questionnaire | 0.359 | 0.266 | 0.186 |
| LifeSatisf_Unadj | NIH Toolbox General Life Satisfaction Survey | Questionnaire | 0.228 | 0.277 | 0^*^ |
| MeanPurp_Unadj | NIH Toolbox Meaning and Purpose Survey | Questionnaire | 0.335 | 0.210 | 0.25 |
| PosAffect_Unadj | NIH Toolbox Positive Affect Survey | Questionnaire | 0.240 | 0.131 | 0.218 |
| Friendship_Unadj | NIH Toolbox Friendship Survey | Questionnaire | 0.472 | 0.077 | 0.472 |
| EmotSupp_Unadj | NIH Toolbox Emotional Support Survey | Questionnaire | 0.232 | 0.078 | 0.232 |
| InstruSupp_Unadj | NIH Toolbox Instrumental Support Survey | Questionnaire | 0.285 | 0.019 | 0.285 |
| SelfEff_Unadj | NIH Toolbox Self-Efficacy Survey | Questionnaire | 0.294 | 0.172 | 0.244 |
| NEOFAC_O | NEO-FFI Openness to Experience | Questionnaire | 0.603 | 0.215 | 0.603 |
| NEOFAC_C | NEO-FFI Conscientiousness | Questionnaire | 0.486 | 0.174 | 0.486 |
| NEOFAC_E | NEO-FFI Extraversion | Questionnaire | 0.477 | 0.322 | 0.31 |
| NEOFAC_A | NEO-FFI Agreeableness | Questionnaire | 0.437 | 0.264 | 0.346 |
| NEOFAC_N | NEO-FFI Neuroticism | Questionnaire | 0.482 | 0.280 | 0.404 |

# This negative twin correlation was corrected to zero for later calculations.

* For these two measures where rDZ was higher than rMZ, the heritability was estimated to be zero.
