# Supplementary material for: Estimating the heritability of psychological measures in the Human Connectome Project dataset
Source: PLoS One. 2020 Jul 9;15(7):e0235860. doi: 10.1371/journal.pone.0235860 (PMC7347217; doi:10.1371/journal.pone.0235860)
Supplement: S2 Table — The Table shows the loadings of each of the 37 measures (rows) onto the 9 factors that we derived (absolute values less than 0.30 were suppressed). (DOCX) [file pone.0235860.s008.docx]

**S2_Table.** Loading matrix for factor analysis. The Table shows the loadings of each of the 37 measures (rows) onto the 9 factors that we derived (absolute values less than 0.30 were suppressed).

| **Loading Matrix** | | | | | | | | | |
| --- | --- | --- | --- | --- | --- | --- | --- | --- | --- |
|  | **Factor** | | | | | | | | |
|  | 1 | 2 | 3 | 4 | 5 | 6 | 7 | 8 | 9 |
| NEOFAC_O |  |  |  |  |  |  |  | .54 |  |
| NEOFAC_C |  |  |  |  |  |  | .56 |  |  |
| NEOFAC_E | .53 |  |  |  |  |  | .32 |  |  |
| NEOFAC_A_corrected |  |  |  |  |  | .74 |  |  |  |
| NEOFAC_N |  | .45 |  |  |  |  | -.50 |  |  |
| PMAT24_A_CR |  |  | .67 |  |  |  |  |  |  |
| PicSeq_Unadj |  |  | .53 |  |  |  |  |  |  |
| CardSort_Unadj |  |  |  |  | .68 |  |  |  |  |
| Flanker_Unadj |  |  |  |  | .72 |  |  |  |  |
| ReadEng_Unadj |  |  | .57 |  |  |  |  | .38 |  |
| ProcSpeed_Unadj |  |  |  |  | .50 |  |  |  |  |
| DDisc_AUC_200 |  |  |  | .81 |  |  |  |  |  |
| DDisc_AUC_40K |  |  |  | .89 |  |  |  |  |  |
| VSPLOT_TC |  |  | .46 |  |  |  |  |  |  |
| SCPT_SEN |  |  |  |  |  |  |  |  |  |
| SCPT_SPEC |  |  | .45 |  |  |  |  |  |  |
| IWRD_TOT |  |  | .37 |  |  |  |  |  |  |
| ListSort_Unadj |  |  | .56 |  |  |  |  |  |  |
| PicVocab_Unadj |  |  | .50 |  |  |  |  | .40 |  |
| ER40_CR |  |  | .37 |  |  |  |  |  |  |
| AngAffect_Unadj |  | .83 |  |  |  |  |  |  |  |
| AngHostil_Unadj |  |  |  |  |  |  |  |  |  |
| AngAggr_Unadj |  |  |  |  |  | -.56 |  |  |  |
| FearAffect_Unadj |  | .96 |  |  |  |  |  |  |  |
| FearSomat_Unadj |  | .63 |  |  |  |  |  |  |  |
| Sadness_Unadj |  | .79 |  |  |  |  |  |  |  |
| Loneliness_Unadj | -.58 |  |  |  |  |  |  |  |  |
| PercHostil_Unadj |  |  |  |  |  |  |  |  | .41 |
| PercReject_Unadj | -.57 |  |  |  |  |  |  |  | .43 |
| PercStress_Unadj |  | .55 |  |  |  |  |  |  |  |
| LifeSatisf_Unadj | .53 |  |  |  |  |  |  |  | .32 |
| MeanPurp_Unadj | .48 |  |  |  |  |  |  |  |  |
| PosAffect_Unadj | .59 |  |  |  |  |  |  |  |  |
| Friendship_Unadj | .84 |  |  |  |  |  |  |  |  |
| EmotSupp_Unadj | .94 |  |  |  |  |  |  |  |  |
| InstruSupp_Unadj | .68 |  |  |  |  |  |  |  |  |
| SelfEff_Unadj |  |  |  |  |  |  | .51 |  |  |
