# Supplementary material for: Estimating the heritability of psychological measures in the Human Connectome Project dataset
Source: PLoS One. 2020 Jul 9;15(7):e0235860. doi: 10.1371/journal.pone.0235860 (PMC7347217; doi:10.1371/journal.pone.0235860)
Supplement: S3 Table — (DOCX) [file pone.0235860.s009.docx]

**Table S3.** List of 9 latent factors with interpretations, twin correlations and heritability estimates.

| Factors | rMZ | rDZ | heritability |
| --- | --- | --- | --- |
| Fac1 positive social relationship | 0.394 | 0.220 | 0.348 |
| Fac2 negative affect | 0.411 | 0.343 | 0.136 |
| Fac3 general intelligence | 0.734 | 0.601 | 0.266 |
| Fac4 impulsivity | 0.529 | 0.360 | 0.338 |
| Fac5 attention and processing speed | 0.473 | 0.161 | 0.473 |
| Fac6 agreeableness | 0.495 | 0.295 | 0.4 |
| Fac7 efficacy and conscientiousness | 0.533 | 0.351 | 0.364 |
| Fac8 language and communication | 0.630 | 0.372 | 0.516 |
| Fac9 competitiveness | 0.292 | 0.136 | 0.292 |
